# Capsules

**Published:** 1874-12

**Authors:** 


*Capsules.*

Many nauseous medicines are now made easy of administration by capsules. A great variety are thus prepared by H. Planten & Son—a reliable house— 224 William St., N. Y. See their card. It is a reliable house

